# Genomics and computational science for virus research

**DOI:** 10.3389/fmicb.2013.00042

**Published:** 2013-03-07

**Authors:** Hironori Sato, Masaru Yokoyama, Hiroyuki Toh

**Affiliations:** ^1^Pathogen Genomics Center, National Institute of Infectious DiseasesTokyo, Japan; ^2^Computational Biology Research Center, National Institute of Advanced Industrial Science and TechnologyTokyo, Japan

RNA viruses are highly mutable, yet changes in genomes and proteins would be restricted by the functional and structural constraints inherent in the survival strategies of viruses in nature. Rapidly evolving technologies in genomics and computational science are now opening up a new avenue for elucidating the real picture of diversity of the organism in nature and for studying the principles underlying the maintenance and change of structures, interactions, and functions of biomolecules. The information is essential for understanding the evolutionary dynamics of virus-host interactions in virological, immunological, and epidemiological phenomena and for rationally developing methods to control RNA viruses. In this Research Topic, we present 17 timely articles, consisting of 5 reviews, 3 mini reviews, 7 original researches, 1 hypothesis & theory, and 1 perspective, all of which underscore the challenges and increasing importance of incorporating the new technologies to study RNA viruses and their impacts on hosts.

## Explorations of viral quasispecies, microRNAs, and antibodyomes in nature

Beerenwinkel et al. (2012) reviewed the challenges and opportunities in inferring the diversity of intra-host virus populations using next-generation sequencing technologies. They discuss the wisdom of reducing artificial errors during sample preparation, existing approaches inferring local and global diversity from sequence data, and successful applications on basic and biomedical studies. Tan Gana et al. (2012) reviewed the latest articles describing cellular and viral microRNAs involved in HIV-1 infection. They describe recent advances in understanding of the biogenesis and functions of the microRNAs in the virus-cell battles and point out roles of the genomics and computational science in obtaining and integrating the information.

Prabakaran et al. (2012) reported on the antibodyomes of 10 healthy individuals obtained by 454 pyrosequencing and bioinformatics analyses. They showed genetic evidence that the antibody subsets with distinct diversity and related to the already-known neutralizing antibodies against the HIV-1, SARS coronavirus, and henipaviruses exist in human IgM repertoires of uninfected individuals. Zhu et al. (2012) reported an antibodyome of an HIV-1-infected individual who produced broadly neutralizing antibodies. Using 454 pyrosequencing, bioinformatics, and functional analyses, they suggested a role of somatic maturation in generating heavy- and light-chain sequences with varied neutralization phenotypes against HIV-1.

## Computational analyses of the 3-D structures, interactions, and evolution of proteins using genetic information

Ode et al. (2012) reviewed the results of molecular dynamic simulations to learn the structural dynamics of proteins in solution. They highlight studies on the structure and function of viral enzymes, virion structures, mechanisms of viral resistance against host immunities and anti-viral drugs, and the development of anti-viral agents. Franzosa et al. (2012) reviewed structural systems biology of interactomes in the host-pathogen relationships. They present existing experimental datasets of the host-pathogen interactome and discuss approaches to obtain structural interactome by integrating the biophysical, functional, and evolutionary information. Miki and Katayama (2012) presented a viewpoint on the *in silico* 3-D structural analysis in virus research. They describe importance of incorporating *in silico* modeling techniques into experimental studies to solve structural problems in their neutralization study of the norovirus.

Bozek et al. (2012) provided *in silico* structural models of capsid proteins of HIV-2 and SIV, which revealed marked differences in the electrostatic potential on the interaction surface and suggested a potential role of electrostatic interactions in the evasion of SIV from the rhesus restriction factor Trim5α. Daiyasu et al. (2012) reported a new application of information theory to the study of the divergent evolution of function of chemokine receptors and their homologs, such as decoy and viral receptors, in which both sequence and structural information are used to identify amino acid positions that might be responsible for evolving their distinct functions. Rusu et al. (2012) provided *in silico* structural models of the Rift Valley fever virus glycoproteins Gn and Gc. The models with the cryo-electron microscopy data allowed the authors to identify four possible arrangements of the glycoproteins in the virion envelope and to indicate how these proteins assemble to form the capsomer base and intercapsomer connections. Yokoyama et al. (2012) provided *in silico* structural models of sapovirus protease docked to its substrate peptides; these models described how this enzyme realizes the functional binding of cleavage sites with distinct sequences and allowed rational identification of the sapovirus protease inhibitors in combination with experimental approaches.

## Analyses of virus-cell interactions, viral replication, and host immune responses

Iwami et al. (2012) reported a mathematical model to quantitatively characterize the viral replication in cell cultures. In their study, the data from two time-course experiments of infections with a cell-free virus stock are used to estimate the half-life of infected cells, viral production rate of an infected cell, and the basic reproductive number. Takemura and Murakami (2012) reviewed structure and function of HIV-1 capsid proteins. They describe the capsid structure in relation to their abilities to form a conical core in a virion or to interact with various cellular proteins that promote or suppress viral replication. Nomura and Matano (2012) reviewed the critical roles of host HLA/MHC-I genotypes in disease progression in primate lentivirus infections. They highlighted studies showing the association of the HLA/MHC-I genotypes with rapid or slow AIDS progression during HIV/SIV persistent infections. Kuroki et al. (2012) reviewed the structural biology of the immunologically intriguing cell surface receptors termed paired receptors. By referencing recent studies of two major structural superfamilies, the immunoglobulin-like and the C-type lectin-like receptors, they described how these receptors discriminate self and non-self ligands to maintain homeostasis in the immune system.

## Analyses of viral transmission network, evolution, and re-emerging diseases

Shiino (2012) reviewed phylodynamic analyses of viral infections, transmission, and evolution in host populations. The author highlighted recent viral researches inferring epidemiologically important parameters, such as infectious clusters, transmission connections, basic reproductive number, and fluctuation of the viral population size. Sasaki et al. (2012) proposed a mathematical model that describes the temporal dynamics of the spread of poliovirus avirulent and virulent strains in a human population; this model was used to estimate the risk of outbreak following the cessation of administration of oral polio vaccine and to predict conditions that significantly increased the outbreak risk.

In summary, these articles present overviews and examples of “Frontiers in Virology” to elucidate viral diversity, protein 3-D structures, virus-host interactions, and evolution of RNA viruses using forefront technologies in genomics and computational science.

## References

[B1] Beerenwinkel N. Gunthard H. F. Roth V. Metzner K. J. (2012). Challenges and opportunities in estimating viral genetic diversity from next-generation sequencing data. Front. Microbiol. 3:329 10.3389/fmicb.2012.00329 22973268 PMC3438994

[B2] Bozek K. Nakayama E. E. Kono K. Shioda T. (2012). Electrostatic potential of human immunodeficiency virus type 2 and rhesus macaque simian immunodeficiency virus capsid proteins. Front. Microbiol. 3:206 10.3389/fmicb.2012.00206 22679444 PMC3367459

[B3] Daiyasu H. Nemoto W. Toh H. (2012). Evolutionary analysis of functional divergence among chemokine receptors, decoy receptors, and viral receptors. Front. Microbiol. 3:264 10.3389/fmicb.2012.00264 22855685 PMC3405870

[B4] Franzosa E. A. Garamszegi S. Xia Y. (2012). Toward a three-dimensional view of protein networks between species. Front. Microbiol. 3:428 10.3389/fmicb.2012.00428 23267356 PMC3528071

[B5] Iwami S. Sato K. De Boer R. J. Aihara K. Miura T. Koyanagi Y. (2012). Identifying viral parameters from *in vitro* cell cultures. Front. Microbiol. 3:319 10.3389/fmicb.2012.00319 22969758 PMC3432869

[B6] Kuroki K. Furukawa A. Maenaka K. (2012). Molecular recognition of paired receptors in the immune system. Front. Microbiol. 3:429 10.3389/fmicb.2012.00429 23293633 PMC3533184

[B7] Miki M. Katayama K. (2012). *In silico* 3D structure analysis accelerates the solution of a real viral structure and antibodies docking mechanism. Front. Microbiol. 3:387 10.3389/fmicb.2012.00387 23133439 PMC3490151

[B8] Nomura T. Matano T. (2012). Association of MHC-I genotypes with disease progression in HIV/SIV infections. Front. Microbiol. 3:234 10.3389/fmicb.2012.00234 22754552 PMC3386493

[B9] Ode H. Nakashima M. Kitamura S. Sugiura W. Sato H. (2012). Molecular dynamics simulation in virus research. Front. Microbiol. 3:258 10.3389/fmicb.2012.00258 22833741 PMC3400276

[B10] Prabakaran P. Zhu Z. Chen W. Gong R. Feng Y. Streaker E. (2012). Origin, diversity, and maturation of human antiviral antibodies analyzed by high-throughput sequencing. Front. Microbiol. 3:277 10.3389/fmicb.2012.00277 22876240 PMC3410596

[B11] Rusu M. Bonneau R. Holbrook M. R. Watowich S. J. Birmanns S. Wriggers W. (2012). An assembly model of rift valley Fever virus. Front. Microbiol. 3:254 10.3389/fmicb.2012.00254 22837754 PMC3400131

[B12] Sasaki A. Haraguchi Y. Yoshida H. (2012). Estimating the risk of re-emergence after stopping polio vaccination. Front. Microbiol. 3:178 10.3389/fmicb.2012.00178 22783231 PMC3390584

[B13] Shiino T. (2012). Phylodynamic analysis of a viral infection network. Front. Microbiol. 3:278 10.3389/fmicb.2012.00278 22993510 PMC3441063

[B14] Takemura T. Murakami T. (2012). Functional constraints on HIV-1 capsid: their impacts on the viral immune escape potency. Front. Microbiol. 3:369 10.3389/fmicb.2012.00369 23087682 PMC3474374

[B15] Tan Gana N. H. Onuki T. Victoriano A. F. Okamoto T. (2012). MicroRNAs in HIV-1 infection: an integration of viral and cellular interaction at the genomic level. Front. Microbiol. 3:306 10.3389/fmicb.2012.00306 22936931 PMC3426883

[B16] Yokoyama M. Oka T. Kojima H. Nagano T. Okabe T. Katayama K. (2012). Structural basis for specific recognition of substrates by sapovirus protease. Front. Microbiol. 3:312 10.3389/fmicb.2012.00312 22973264 PMC3433708

[B17] Zhu J. O'Dell S. Ofek G. Pancera M. Wu X. Zhang B. (2012). Somatic populations of PGT135-137 HIV-1-neutralizing antibodies identified by 454 pyrosequencing and bioinformatics. Front. Microbiol. 3:315 10.3389/fmicb.2012.00315 23024643 PMC3441199

